# Telomeric DNA induces apoptosis and senescence of human breast carcinoma cells

**DOI:** 10.1186/bcr1646

**Published:** 2007-01-26

**Authors:** Mina Yaar, Mark S Eller, Izabela Panova, John Kubera, Lee Hng Wee, Kenneth H Cowan, Barbara A Gilchrest

**Affiliations:** 1 Department of Dermatology, Boston University School of Medicine, Albany Street Boston, MA 02118-2394, USA; 2 Department of Pathology and Laboratory Medicine, Boston University School of Medicine, Albany Street, Boston, MA 02118-2394, USA; 3 Cancer Center, Boston University School of Medicine, Albany Street, Boston, MA 02118-2394, USA; 4 Eppley Institute for Research in Cancer and Allied Diseases, University of Nebraska Medical Center, Omaha, NE, 68198-6805, USA

## Abstract

**Introduction:**

Cancer is a leading cause of death in Americans. We have identified an inducible cancer avoidance mechanism in cells that reduces mutation rate, reduces and delays carcinogenesis after carcinogen exposure, and induces apoptosis and/or senescence of already transformed cells by simultaneously activating multiple overlapping and redundant DNA damage response pathways.

**Methods:**

The human breast carcinoma cell line MCF-7, the adriamycin-resistant MCF-7 (Adr/MCF-7) cell line, as well as normal human mammary epithelial (NME) cells were treated with DNA oligonucleotides homologous to the telomere 3' overhang (T-oligos). SCID mice received intravenous injections of MCF-7 cells followed by intravenous administration of T-oligos.

**Results:**

Acting through ataxia telangiectasia mutated (ATM) and its downstream effectors, T-oligos induced apoptosis and senescence of MCF-7 cells but not NME cells, in which these signaling pathways were induced to a far lesser extent. In MCF-7 cells, experimental telomere loop disruption caused identical responses, consistent with the hypothesis that T-oligos act by mimicking telomere overhang exposure. *In vivo*, T-oligos greatly prolonged survival of SCID mice following intravenous injection of human breast carcinoma cells.

**Conclusion:**

By inducing DNA damage-like responses in MCF-7 cells, T-oligos provide insight into innate cancer avoidance mechanisms and may offer a novel approach to treatment of breast cancer and other malignancies.

## Introduction

Breast cancer is the leading cancer among women [1]. Therapy for advanced disease relies largely on chemotherapy and radiation therapy (reviewed in [2-5]), both of which have significant host toxicity and themselves promote mutations, fueling disease progression and treatment resistance.

Despite progress in treatment modalities for patients with metastatic disease, median survival is two to three years [6] and treatment with single or multiple chemotherapeutic agents is invariably toxic to normal proliferating tissues [6]. Promising novel therapies directed at specific malfunctioning proteins, including anti-receptor tyrosine kinase antibodies [7], will no doubt greatly improve treatment outcomes. Still, to date, despite substantial variability in patient survival, metastatic breast cancer is almost invariably fatal (reviewed in [5,8]).

Recent work from our laboratory [9-15] suggests the existence of innate inducible cellular responses, initiated by exposure of the telomere repeat sequence TTAGGG at the time of telomere loop disruption and mediated through multiple redundant signaling pathways, that act to retard cancer initiation and progression by selectively eliminating cells with acute severe DNA damage or after multiple rounds of cell division [16]. These responses fail to occur in malignant cells, despite their loss of normal check points and often gross DNA abnormalities, but can be triggered by providing cells or tissues with DNA oligonucleotides homologous to the telomere 3' overhang (T-oligos) [9,13,17]. A large number of partially and completely homologous sequences have been found effective [9-14,17,18], with longer sequences generally having a higher molar efficacy than shorter ones, at least in the 2 to 20 base range [18,19].

Telomeres exist in a loop structure that is stabilized by telomeric repeat binding factor (TRF)2 [12]. Disruption of the loop by a dominant negative construct (TRF2^DN^) leads to apoptosis of certain mammalian cells [20] and senescence of others [10,20], mediated at least in part through the ataxia telangiectasia mutated (ATM) kinase, with subsequent p53 activation [10,20-22], demonstrating that telomere loop disruption in some way constitutes a DNA damage signal. We have shown that exposure of cells to T-oligos leads to dose-dependent DNA damage responses in both normal and malignant cells, mediated at least in part through the ATM kinase and its effector proteins p53, p73, p95/Nbs1, and E2F1 [9-11,13]. Moreover, in several human malignant cell types, including lymphoma, melanoma, fibrosarcoma, and squamous cell carcinoma, T-oligos induce differentiation, apoptosis and/or senescence [9,13,17], and strikingly reduce tumor burden in a mouse xenograft model of human melanoma [13]. These cellular responses occur without affecting the cells' own telomeres [9,15], and appear to be far more pronounced in malignant cells than in their normal counterparts [13,20]. T-oligos have also been shown to activate p53 and to enhance DNA repair rate in intact murine skin and human skin explants [12,23] and to decrease mutation rate and photocarcinogenesis after UV irradiation in mice [12]. Because the telomere overhang, repeats of TTAGGG, is concealed within the proximal duplex telomere except at times of experimental opening of the telomere loop, or presumptively following physiological stimuli such as acute DNA damage or critical telomere shortening during serial cell passage or transiently at times of DNA replication [9,10,15], and because T-oligos rapidly accumulate in the nucleus [9,19], we hypothesize that T-oligos mimic exposure of the overhang and are interpreted by the cell as indicating that these anti-cancer responses should be initiated.

DNA damage characteristically leads to phosphorylation of ATM on serine 1981 [24,25]. ATM then phosphorylates p53 on serine-15 [26,27], p95/Nbs1 on serine 343 [21] and histone H2AX on serine 139 [28,29]. DNA damage also induces E2F1, a process dependent on both the ATM and the ATM-Rad3-related (ATR) kinases [30]. As well, E2F1 transcriptionally upregulates p73 [31], a structural and functional homolog of p53 that accumulates in response to DNA damage. In normal human fibroblasts, prolonged T-oligo treatment induces irreversible growth arrest and p53 phosphorylation as well as induction of p16, p53, p21, p95/Nbs-1 and senescence-associated (SA) β galactosidase (β-gal) activity [10], mimicking both serial passage to senescence (reviewed in [10,32]) and telomere loop disruption by TRF2^DN ^[10].

To further test our hypothesis regarding the T-oligo mechanism of action [14,16] and to quantify T-oligo therapeutic effects, we studied the breast carcinoma line MCF-7 and the same line after being rendered resistant to adriamycin. We also compared the T-oligo effect on MCF-7 cells to its effect on normal mammary epithelial (NME) cells. In cancerous cells, T-oligo induces apoptosis and senescence of MCF-7 (p16-null) and adriamycin resistant MCF-7 (p16 null, non-functional p53, multiple drug resistant (MDR)-1 positive) breast cancer cells, but these effects are significantly less prominent in NME cells. We also demonstrate that systemic administration of T-oligos significantly prolongs the survival of severe combined immunodeficient (SCID) mice in a model of metastatic breast cancer.

## Materials and methods

### Materials

Two DNA oligonucleotides were designed, one homologous to the telomere overhang (T-oligo, GTTAGGGTTAG) and one complementary to this sequence (Control-oligo, CTAACCCTAAC) (Midland Certified Reagent, Midland, TX, USA). Oligonucleotides were resuspended in H_2_O and subsequently diluted in culture medium for a final concentration of 40 μM. In all experiments, T-oligo was added only once. Cisplatin (Sigma, St Louis, MO, USA) was used at a final concentration of 10 μM and ICI 182,780 (Tocris Cookson Inc., Ellisville, MI, USA) at 100 nM. For *in vivo *experiments, GTTAGGGTTAG and a 16-base T-oligo, GGTTAGGTGTAGGTTT, were obtained from Trilink (San Diego, CA, USA). For immunofluorescent studies, the 11mer GTTAGGGTTAG was tagged with fluorescein phosphoramidite (FAM) on the 3' end (Midland).

For *in vivo *studies, the intravenous dose of the 16-mer was 60 versus 120 nmoles of the 11mer, doses calculated as equivalent to 20 μM versus 40 μM when diluted in the mouse blood volume (estimated at 1.5 ml) immediately after injection.

### Cell culture

MCF-7 (estrogen receptor-α positive, p16 null, wild-type p53) cells were a gift of Dr Brown (Division of Molecular and Cellular Oncology, Department of Medical Oncology, Dana-Farber Cancer Institute Boston, MA, USA). Cells were maintained in MEM supplemented with 200 mM L-glutamine and Earle's BSS adjusted to contain 1.5 g/l sodium bicarbonate, 0.1 mM non-essential amino acids and 1 mM sodium pyruvate and supplemented with 0.01 mg/ml bovine insulin, and 10% FBS. Adriamycin resistant MCF-7 cells (Adr/MCF-7) [33] were maintained in the above medium supplemented with 1.5 μg/ml doxorubicin (Sigma Co., St Louis, MO, USA). NME cells were obtained from Clonetics (Walkersville, MD, USA) and maintained in Mammary Epithelial Cell Growth Medium supplemented with brain pituitary extract, human epidermal growth factor, hydrocortisone, insulin, gentamicin and amphotericin B according to the manufacturer's instructions. FBS (5%) was added at the time of definitive experiments.

### Immunofluorescent microscopy

Cells were provided 40 μM of FAM-labeled oligonucleotides. After 60 minutes, medium was removed and cells were fixed in 3.7% formaldehyde (10 minutes) followed by 0.1% Triton X-100, 2 mM EDTA (pH 7.4), 0.5 mg/ml RNase A and 0.375 mM propidium iodide in PBS (10 minutes). Intracellular T-oligo was visualized using a multicolor fluorescent microscope (Nikon), providing excitation at 488 nm and 583 nm and detection at 503 to 530 nm (green-FAM channel) and 560 nm (red-propidium iodide channel) at constant settings for paired dishes in each experiment. Digital images were obtained.

### Western blot analysis and antibodies

Western blot analysis was performed as described [34]; 50 to 100 μg protein/lane were processed. Antibody reactions were performed with mouse monoclonal or rabbit polyclonal antibodies: anti-p53 (DO-1, Ab-6) and anti p73 (Ab-4) (Oncogene Research Products, Cambridge, MA, USA), anti-phospho-p53 ((Ser-15), 9284), anti-phospho-Rb (Ser 795), anti-phospho-p95/Nbs1 (Ser 343) and anti-phospho-H2AX (Ser 139) (Cell Signaling Technology, Beverly, MA, USA), anti-p21/SDI1 (C24420; Transduction Laboratories, Lexington, KY, USA), anti-phospho-ATM (Ser 1981; Rockland, Gilbertsville, PA, USA) and anti-E2F-1 (Santa Cruz Biotechnology, Santa Cruz, CA, USA). Antibody binding was detected by the ECL detection kit (Amersham, Piscatawya, NJ, USA), followed by autoradiography (Kodak X-Omatic AR).

### Densitometric analysis

Autoradiograms were scanned into a computer (PC Dell™). Band intensity was determined after background subtraction using BIO-RAD Gel Doc 1000/2000 imaging densitometer (BIO-RAD, Hercules, CA, USA).

### Cell yield

After removing the medium and rinsing with PBS, cultures were incubated in 0.25% trypsin at 37°C and cell yields were determined using a particle counter (Coulter Corp., Miami, FL, USA).

### Cell cycle analysis

Cells from duplicate cultures were collected, fixed in ethanol, stained with propidium iodide [10] and analyzed for DNA content using FACScan analysis (Becton Dickinson, San Jose, CA, USA). The distribution of cells between different phases of the cell cycle was determined using the CELLQUEST program (Becton Dickinson, San Jose, CA).

### Apoptosis

#### TUNEL analysis

Apoptosis was determined as described [35]. Cells were collected, fixed in 4% paraformaldehyde, permeabilized with 0.1% Triton X-100 in 0.1% sodium citrate, then incubated with fluorescein-tagged dUTP in the presence of terminal deoxynucleotidyl transferase (Boehringer Mannheim Biochemicals, Mannheim, Germany) and cellular fluorescence was analyzed by flow cytometry (Becton Dickinson).

#### ELISA

DNA fragmentation as a measure of apoptosis was determined using the cell death detection ELISA^PLUS ^kit and following the manufacturer's instructions (Roche Diagnostics, Indianapolis, IN, USA) [36]. Briefly, MCF-7 and NME cells were stimulated with T-oligo GTTAGGGTTAG (40 μM) or diluent and cells were harvested 96 hours after stimulation. Cells (1 × 10^4^) were used as the antigen source in a sandwich ELISA assay with a primary anti-histone antibody coated to the microtiter plate and a secondary anti-DNA antibody coupled to peroxidase.

### BrdU incorporation assay

MCF-7 and NME cells cultured on Permanox chamber slides were treated with T-oligo (GTTAGGGTTAG, 40 μM) or diluent alone for seven days and then were re-fed with medium lacking oligonucleotides. DNA synthesis was assayed using the 5-bromo-2'-deoxyuridine (BrdU) Labeling and Detection Kit II (Roche Molecular Biochemicals, Indianapolis, IN, USA) following the manufacturer's protocol. Briefly, cells were labeled for 1 hour with BrdU, fixed and incubated with anti-BrdU monoclonal antibody. After incubation with anti-mouse Ig-alkaline phosphatase, the color reaction was detected and pictures of several representative fields were obtained. Four different fields were analyzed and percent positive cells was calculated. For NME cells that grow in colonies, BrdU labeled cells were determined throughout the colonies in subconfluent cultures.

### TRF2^DN ^transfection

The myc-tagged adenovirus (Ad)TRF2^DN ^expression vector was the gift of Dr de Lange (The Rockefeller University, New York, NY, USA). We plated 3.6 × 10^3 ^cells/cm^2^. One day later, cultures were supplemented with 7 × 10^10 ^viral particles/ml of either AdTRF2^DN ^or Ad-green fluorescent protein (GFP; control). The next day, medium was changed and the transfection was repeated. Cells were collected at different intervals.

### SA β-galactosidase staining

Cells were treated once with diluent, T-oligo or control oligo for one week, fixed for 3 to 5 minutes, and incubated at 37°C (no CO_2_) overnight with fresh SA β-gal stain solution as described [32].

### Animal model

Studies were performed in accordance with Boston University's animal review board. Nine-week slow release 17-β-estradiol pellets (#SE121, Innovative Research of America, Sarasota, FL, USA) were injected subcutaneously into six-week-old SCID mice (Fox-Chase Cancer Center, Philadelphia, PA, USA). One day later, 2 × 10^6 ^MCF-7 cells were injected intravenously into the tail vein of each mouse. Then, 3 days later, groups of 5 mice each began receiving blinded intravenous injections: the 11-base T-oligo (120 nmoles/injection, 23 mg/kg, 100 μl solution in Hank's buffered salt solution (HBSS) assuming a 1.5 ml blood volume distribution) twice daily 5 days/week for 1 week; the 16-base T-oligo (60 nmoles/injection, 15 mg/kg); or diluent alone (HBSS). The same regimen was repeated on day 31, after the first mouse died. Animals were inspected and weighed twice weekly and euthanized when they lost ≥15% of their body weight within 7 days.

### Statistical analysis

Differences in cell yields, in the level of cytoplasmic DNA-histone complexes and in BrdU incorporation were determined using General Linear Model with *post hoc *analysis (SPSS Inc., statistical package version 10.0, Chicago, IL, USA). Differences in S phase distribution were determined by one way ANOVA with *post hoc *analysis. Differences in SCID mice survival were determined by the Kaplan-Meier survival test (SPSS Inc.).

## Results

### T-oligo localizes to nuclei

**Figure 1:**
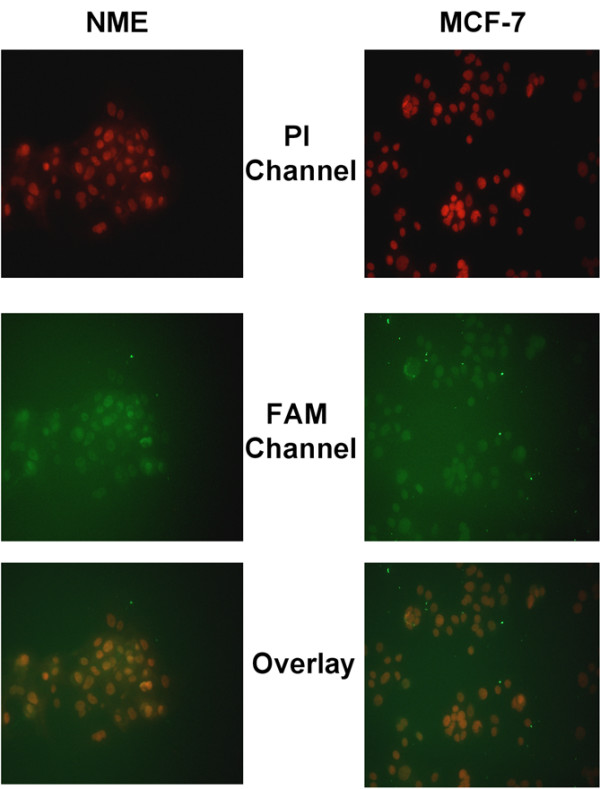
T-oligos localize to nuclei. MCF-7 and normal mammary epithelial (NME) cells were supplemented with fluorescein phosphoramidite (FAM) labeled GTTAGGGTTAG and uptake was determined 30 to 60 minutes after supplementation. Multicolor fluorescent microscopy showed that FAM-labeled T-oligos accumulated in the nucleus as determined by colocalization of propidium iodide (PI) nuclear staining (red) with FAM (green), producing an orange color. As expected, no green fluorescence was observed in control, diluent-treated cells.

To determine if oligonucleotides comparably enter MCF-7 and NME cells, cultures were provided FAM-labeled GTTAGGGTTAG and, within 30 to 60 minutes, T-oligo localization was examined using a multicolor fluorescent microscope. As previously observed in multiple normal and malignant cell types [9,19], T-oligo rapidly localized to the nuclei comparably well in both cell types (Figure 1) and the majority of nuclei were positive. No green fluorescence was observed in control, diluent-treated cells (data not shown).

### T-oligo inhibits cell growt

**Figure 2:**
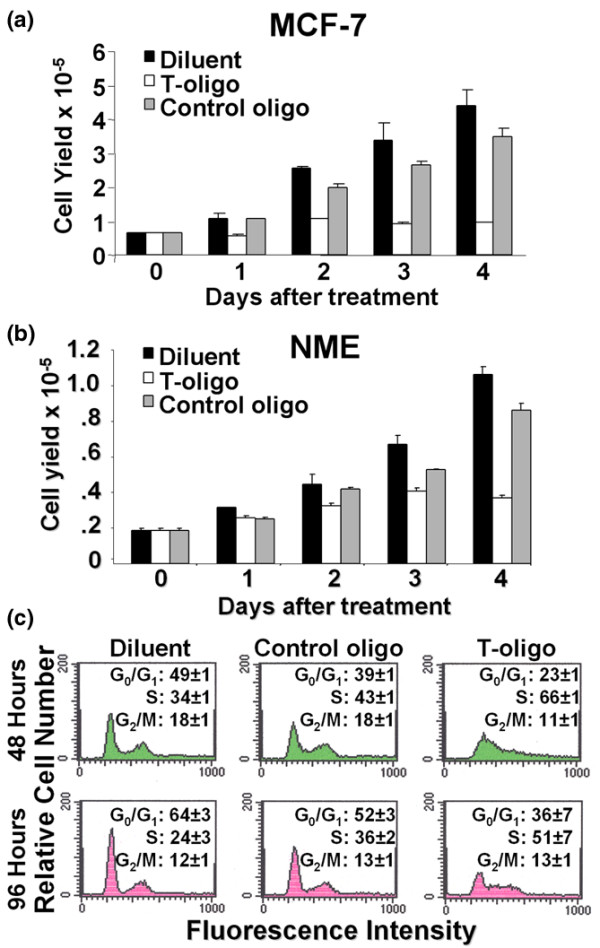
T-oligo decreases cell yield and induces S phase arrest in MCF-7 cells. Preconfluent cultures of **(a) **MCF-7 cells and **(b) **normal mammary epithelial (NME) cells were treated with 40 μM of GTTAGGGTTAG (T-oligo), CTAACCCTAAC (control oligo) or diluent alone and cell yields were determined at different intervals after oligonucleotide addition. Compared to controls, within 4 days T-oligo decreased cell yields 87 ± 9% (MCF-7; *p *< 0.04, *n *= 4) and 65.5 ± 0.01% (NME; *p *< 0.03, *n *= 2). **(c) **MCF-7 cells were treated as above and cells were collected for up to 96 hours for FACScan analysis after a single treatment at time 0. Averages and standard deviations were determined from duplicate samples. T-oligo increased the percent of cells in the S phase of the cycle as early as 48 hours after stimulation (*p *< 0.001, *n *= 2) and for at least 96 hours after stimulation (*p *< 0.04, *n *= 2).

To determine the effect of T-oligo (11mer, 40 μM) on cell growth, we examined MCF-7 and NME cell yields up to 96 hours after a single supplementation of T-oligo (Figure 2). A decrease in MCF-7 yield was observed as early as 1 day after treatment. Diluent and control-oligo treated cells displayed exponential growth versus extremely little or no growth of T-oligo-treated cells. T-oligo decreased MCF-7 yields by 87 ± 9% within 4 days when the experiment was terminated (Figure 2a). Similarly, T-oligo inhibited NME proliferation, although the magnitude of the effect was smaller, with yields decreased by only 65.5 ± 0.01% after 4 days (Figure 2b). Modest but statistically less inhibition was observed in control oligo-treated cells.

### T-oligo induces S phase cell cycle arrest of MCF-7 cells

To determine if the T-oligo effect on MCF-7 yields is due, at least in part, to an effect on the cell cycle, MCF-7 cells were treated as above (Figure 2c). A single supplementation of T-oligo produced S phase cell cycle arrest, as reported previously for other cell types [9,13,17,37] with 66 ± 1% and 51 ± 7% of cells arrested in S phase at 48 and 96 hours, respectively. As before, control oligo had a far lesser effect.

### T-oligo induces apoptosis of MCF-7 cells

**Figure 3:**
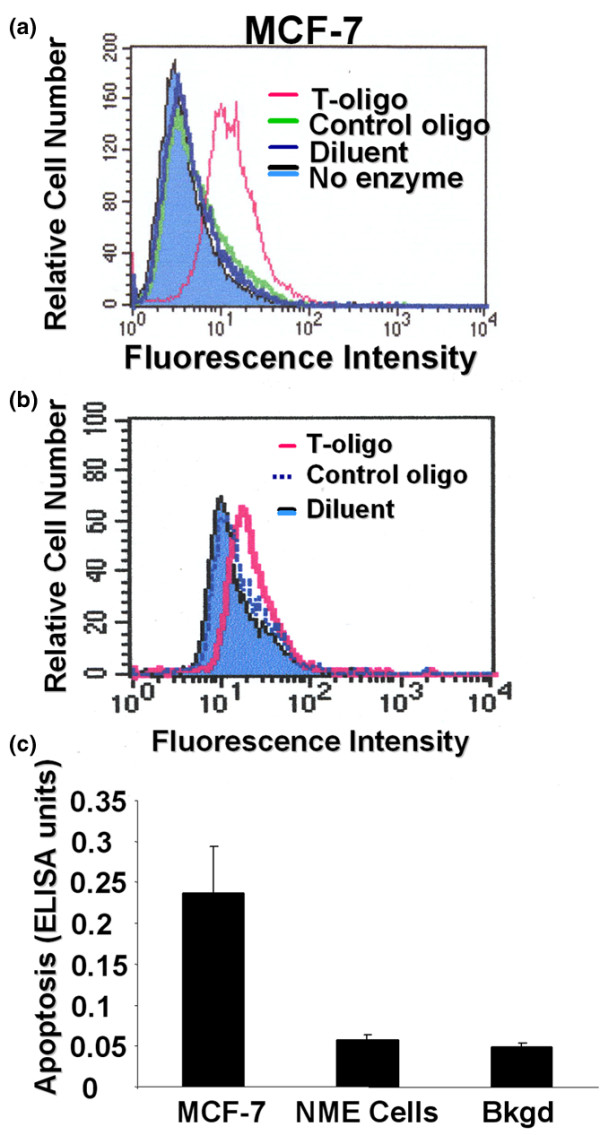
T-oligo induces apoptosis of MCF-7 cells. MCF-7 and normal mammary epithelial (NME) cells were treated as above. Apoptosis was determined by TUNEL analysis and by the level of cytoplasmic DNA-histone complexes 96 hours after a single treatment at time 0. **(a) **MCF-7 cells underwent apoptosis as observed by the prominent shift in fluorescent peak. **(b) **In contrast, there was only a minimal shift in the fluorescent peak of NME cells. **(c) **There is a significant increase in the levels of cytoplasmic DNA-histone complexes in T-oligo-treated MCF-7 cells compared to diluent-treated MCF-7 cells (*p *< 0.02, *n *= 3). In contrast, DNA-histone complexes in NME cells were not significantly increased above the background (Bkgd) level (p = 0.89).

To determine if the T-oligo effect on cell yields in MCF-7 and NME cells is due to apoptosis in addition to S phase arrest, cultures were stimulated with T-oligo as above and TUNEL assays were performed and levels of cytoplasmic DNA-histones were determined 96 hours after stimulation (Figure 3). While in MCF-7 cells T-oligos induced substantial apoptosis, as shown by the prominent shift in the fluorescent peak (Figure 3a), in NME cells there was less of a shift (Figure 3b). In addition, the level of cytoplasmic DNA-histone complexes was significantly higher (*p *< 0.03) in T-oligo-treated MCF-7 cells than in NME cells (Figure 3c), in which the level of cytoplasmic DNA-histone complexes was identical to the background level (*p *= 0.9), showing that T-oligo induces significantly more apoptosis in MCF-7- than in NME cells, thus accounting for the greater reduction in cell yields.

### T-oligo induces senescence of MCF-7 cells

**Figure 4:**
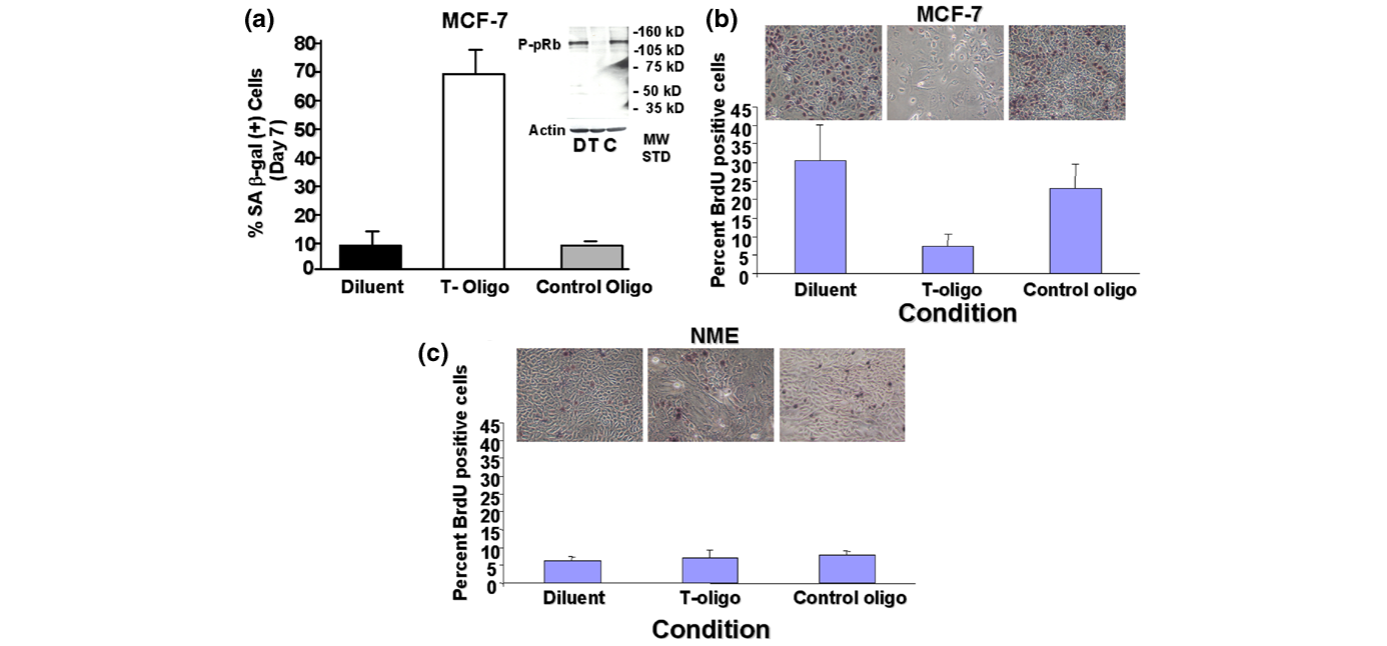
T-oligo induces senescence of MCF-7 cells. **(a,b) **MCF-7 and **(c) **normal mammary epithelial (NME) cells were treated with T-oligo once for seven days and then were either fixed and stained for senescence-associated (SA) β-galactosidase (β-gal) activity (a), or were supplemented with fresh medium lacking oligonucleotides and 5-bromo-2'-deoxyuridine (BrdU) incorporation (b,c) and retinoblastoma protein phosphorylation (inset) were determined. (a) In MCF-7 cells, T-oligo induced senescence of 61.3 ± 7.7% cells, compared to diluent and control oligo in which 6.3 ± 3.8% and 12 ± 2% of the cells, respectively, were senescent, as determined by SA β-gal activity (*p *< 0.015). (b) In MCF-7 cells, after T-oligo removal and provision of medium without T-oligo, only 7.4 ± 3.3% of cells displayed BrdU incorporation compared to 30.4 ± 9.8% and 23 ± 6.4% of diluent and control oligo pretreated cells, respectively (*p *< 0.05). (c) In NME cells, BrdU incorporation did not differ between T-oligo and diluent-treated cultures (7.1 ± 2.2% versus 6.3 ± 1.1% and 7.8 ± 1.2%, respectively; *p *= 0.5). Inset: MCF-7 cells were treated as above. Despite supplementation of fresh medium lacking T-oligo, no phosphorylation of retinoblastoma protein (pRb) was detected in T-oligo pretreated cultures. C, control-oligo; D, diluent; T, T-oligo.

To determine the fate of the remaining non-apoptotic cells, we investigated whether T-oligo induces senescence. Cells were treated once at time 0 with T-oligo or diluent alone and, after seven days, were fixed and stained for SA β-gal activity, a marker for senescence in at least some cell types [32]. Paired cultures were supplemented after seven days with fresh medium lacking oligonucleotides or diluent. BrdU incorporation and retinoblastoma protein phosphorylation (MCF-7 only) were determined after an additional 24 hours. In MCF-7 cells, T-oligo induced senescence of 61.3 ± 7.7% cells, compared to diluent and control oligo, which induced senescence in 6.3 ± 3.8% and 12 ± 2% of the cells, respectively, as determined by SA β-gal activity (*p *< 0.015; Figure 4a).

Similarly, after provision of medium without T-oligo, BrdU incorporation in MCF-7 cells significantly differed between the cultures previously treated with T-oligo (7.4 ± 3.3%) and those previously treated with diluent (30.4 ± 9.8%) or control oligo (23 ± 6.4%) (p < 0.05), consistent with T-oligo induced senescence (Figure 4b). There was no statistically significant difference between diluent and control oligo pre-treated cultures (p = 0.5). Conversely, in NME cells, BrdU incorporation between cultures previously treated with T-oligo versus diluent alone and control oligo did not differ, displaying 7.1 ± 2.2%, 6.3 ± 1.1% and 7.8 ± 1.2% BrdU-positive cells, respectively (p = 0.5), suggesting that NME cells do not undergo senescence, at least not within 7 days after T-oligo treatment (Figure 4c).

Finally, it is well established that, in senescent cells, retinoblastoma protein (pRb) remains in a hypophosphorylated state despite growth stimulation [38]. To further examine the senescene-like effect of T-oligo, we determined pRb phosphorylation in T-oligo treated MCF-7 cells versus controls. T-oligo treated MCF-7 cells failed to phosphorylate pRb despite fresh medium supplementation, while diluent or control-oligo treated cultures displayed strong pRb phosphorylation (Figure 4, inset). These studies demonstrate that T-oligo induces senescence in the remaining MCF-7 cells, those that have not undergone apoptosis, but not in NME cells.

### T-oligo and TRF2^DN ^activate cell cycle and apoptosis regulatory proteins

**Figure 5:**
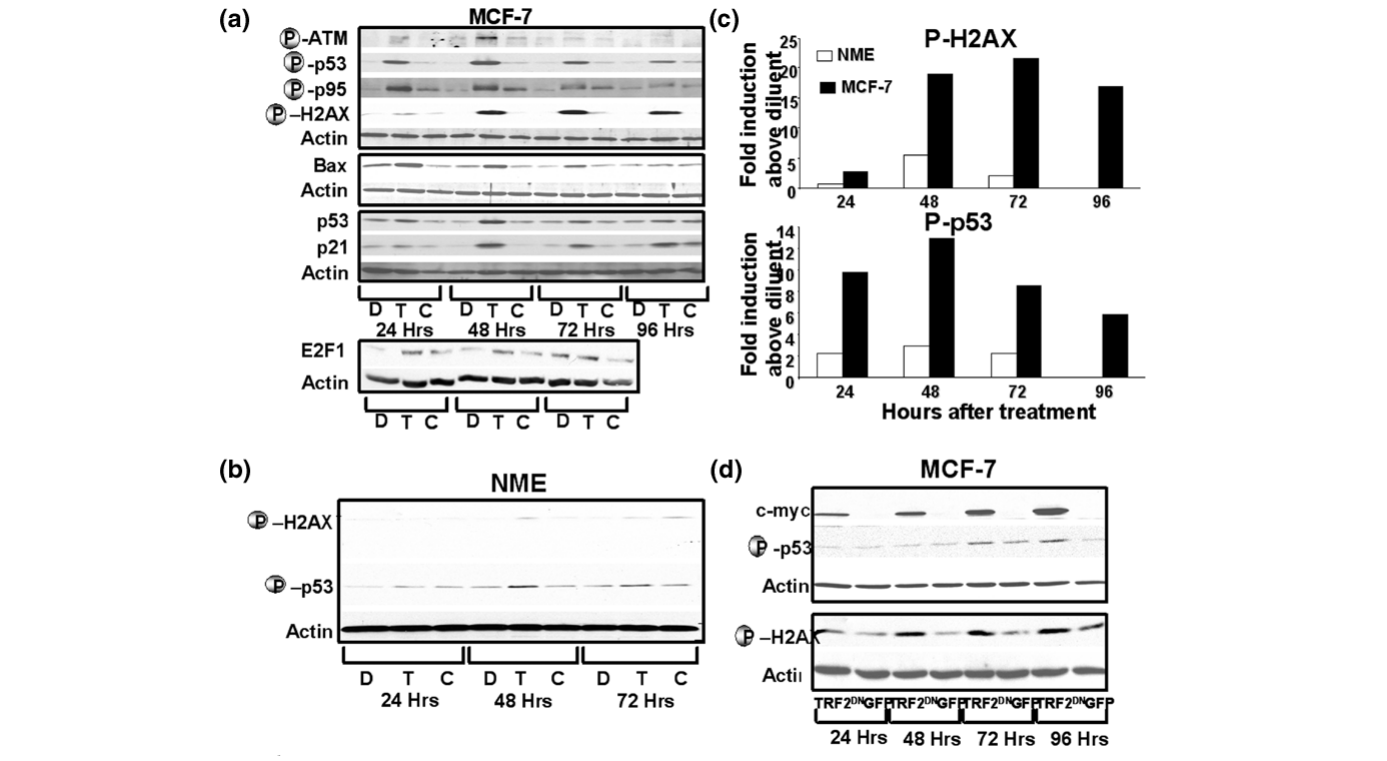
T-oligo and telomeric repeat binding factor dominant negative construct (TRF2^DN^) activate cell cycle- and apoptosis-regulatory proteins in breast cancer cells. **(a) **Compared with control oligo (C) and diluent (D) alone, T-oligo (T) substantially induced the phosphorylation of p53 (Ser15), p95/Nbs1 (Ser343) and H2AX (Ser139) and the level of E2F1. In MCF-7 cells, T-oligo also induced the phosphorylation of ataxia telangiectasia mutated (ATM; Ser1981) and the levels of p53 and the p53-dependent p21 and BAX proteins. **(b) **Compared with control oligo (C) and diluent (D), T-oligo (T) only slightly induced the phosphorylation of p53 and H2AX in normal mammary epithelial (NME) cells. **(c) **Graphic representation of band intensity as fold induction above diluent. **(d) **adenovirus (Ad)TRF2^DN^, but not the control Ad-green fluorescent protein (GFP) vector, induced the phosphorylation of H2AX and p53 in MCF-7 cells. c-myc expression confirms successful transfection of the cells with AdTRF2^DN^. Actin serves as a loading control. One of three representative experiments is shown.

MCF-7 and NME cells were treated as above and total cellular proteins were harvested at different intervals after stimulation. As determined by protein phsophorylation, T-oligo activated the ATM kinase, known to be activated by DNA damage or critically short telomeres [39-41]. Similarly, T-oligo activated (phosphorylated) the downstream effector proteins p53 (serine 15), p95/Nbs1 (serine 343) and H2AX (serine 139) (Figure 5a). T-oligo also induced the levels of E2F1, albeit more rapidly and transiently, and induced the level of the p53-dependent downstream apoptosis effector protein BAX [42] and p21 [43] (Figure 5a).

In contrast, although T-oligo phosphorylated p53 and H2AX in NME cells, the magnitude of these inductions was smaller in NME cells (≤3-fold and ≤6-fold, respectively, at 24 to 72 hours) compared to MCF-7 cells (up to >12-fold and >21-fold, respectively, through at least 96 hours) (Figure 5b,c).

To further substantiate our hypothesis that T-oligo treatment mimics physiological exposure of the 3' telomere overhang following telomere loop disruption [9-11,13,15,17], we introduced TRF2^DN ^[20] into MCF-7 cells, a manipulation known to disrupt the telomere loop and to lead to exposure and digestion of the 3' overhang [15,17,20]. AdTRF2^DN^, but not a control AdGFP vector, induced γ-H2AX and p53 in MCF-7 cells over the same time course as T-oligo (Figure 5d), supporting our hypothesis.

### T-oligo effect on MCF-7 cells is comparable to that of cisplatin and ICI 182,780

**Figure 6:**
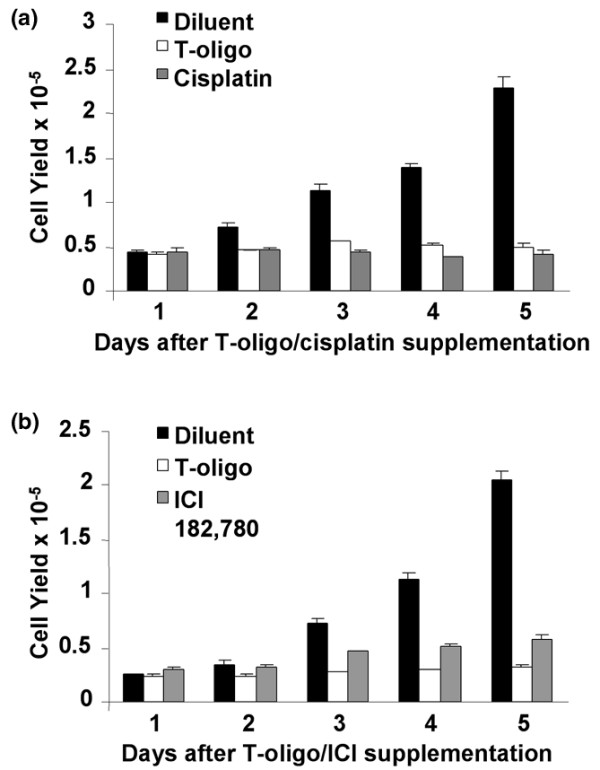
T-oligo effect on MCF-7 cells is comparable to that of cisplatin and ICI 182,780. MCF-7 cells were treated with cisplatin (10 μM), the anti-estrogen ICI 182,780 (100 nM) or T-oligo (40 μM) once and cell yields were determined at different intervals. **(a) **Within 5 days cisplatin decreased MCF-7 yield by 82 ± 0.1% and T-oligo did so by 79 ± 0.02% compared to diluent. The difference between the T-oligo and cisplatin effects was not significant (*p *= 1.0). **(b) **Compared to diluent, ICI 182,780 or T-oligo caused decreases in cell yield of MCF-7 cells of 72 ± 0.1% and 84 ± 0.02%, respectively. T-oligo was significantly more effective than ICI 182,780 (*p *< 0.005).

To compare the T-oligo effect to that of a recognized anti-breast cancer chemotherapeutic agent, cisplatin [44-48], and to an anti-estrogen frequently used to treat estrogen receptor positive breast cancers [49-51], MCF-7 cells were treated with customary doses of cisplatin (10 μM), the anti-estrogen ICI 182,780 (100 nM) or T-oligo (40 μM) as above (Figure 6). Within 5 days after a single treatment, compared to diluent, cisplatin decreased MCF-7 yield by 82 ± 0.1% and T-oligo did so by 79 ± 0.02%, an insignificant difference (*p *= 1.0; Figure 6a). Similarly, compared to diluent, within 5 days after a single treatment with ICI 182,780 or T-oligo, cell yield decreased by 72 ± 0.1% and 84 ± 0.02%, respectively (Figure 6b). Interestingly, in this comparison, T-oligo was significantly more effective than the anti-estrogen ICI 182,780 (*p *< 0.005).

### T-oligo affects adriamycin-resistant breast cancer cells

Multidrug resistance develops in cancer cells as a result of overexpression of a P-glycoprotein MDR-1 that acts as an efflux pump for various anticancer drugs and also inhibits apoptosis, thus reducing the responsiveness of cancer cells to chemotherapeutic agents [52]. Thus, agents that are effective in such non-responsive cells would serve an important role as a second generation anti-cancer treatment option.

**Figure 7:**
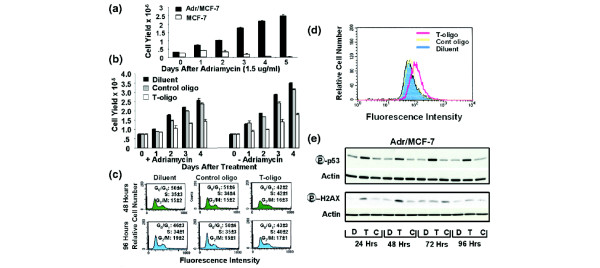
T-oligo affects adriamycin resistant breast cancer cells. **(a) **Unlike MCF-7 cells, Adr/MCF-7 cells proliferate in the presence of adriamycin. **(b) **Supplementation with T-oligo on day 0 decreased Adr/MCF-7 cell yields in both the presence and absence of adriamycin for at least 96 hours (58% ± 14%, *p *< 0.03, *n *= 4). **(b) **T-oligo also increased the percent of cells in the S phase within 48 hours and through 96 hours (*p *< 0.04, *n *= 2) and **(d) **induced apoptosis as determined by TUNEL analysis at 96 hours. **(e) **T-oligo induced the phosphorylation of p53 and H2AX comparably to MCF-7 cells (Figure 5a). C, control-oligo; D, diluent; T, T-oligo.

To determine if T-oligo is effective in MDR-1-positive cells, adriamycin-resistant MCF-7 cells (Adr/MCF-7), known to express high MDR-1 levels [53], were used after confirmation of persistent high expression of the P-glycoprotein (data not shown). Unlike the parental MCF-7, Adr/MCF-7 cells proliferate in the presence of adriamycin (Figure 7a). Supplementation with T-oligo on day 0 decreased Adr/MCF-7 cell yields in both the presence and absence of adriamycin (*p *< 0.03) by 58 ± 14% through at least 96 hours (Figure 7b). Also, compared to diluent and control oligo, T-oligo increased the percent of cells in the S phase within 48 hours through 96 hours (*p *< 0.04; Figure 7c) and induced apoptosis, as determined by the shift in the fluorescent peak observed in the TUNEL analysis at 96 hours (Figure 7d). T-oligo-induced phosphorylation of p53 and H2AX in Adr/MCF-7 cells was also comparable to that in parental MCF-7 cells (Figure 7e).

### T-oligo increases survival of mice injected intravenously with MCF-7 cells

**Figure 8:**
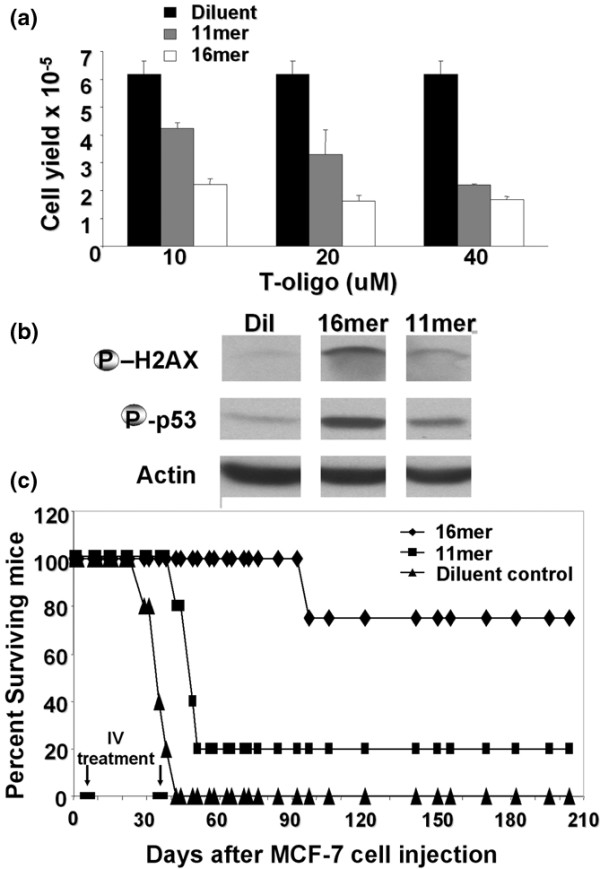
T-oligo increases survival of mice injected intravenously with MCF-7 cells. **(a) **MCF-7 cells received fresh serum-containing medium supplemented with increasing concentrations (10 to 40 μM) of the 11mer or 16mer T-oligos or diluent alone and were harvested at 96 hours. At 10 μM and 20 μM, the 16mer inhibited MCF-7 yield substantially more than the 11mer, and 20 μM 16mer reduced cell yields comparably to 40 μM 11mer. **(b) **MCF-7 cells were supplemented with 11mer or 16mer T-oligos (20 μM) or diluent (Dil) alone. Within 48 hours both T-oligos induced H2AX and p53 phosphorylation, but this occurred to a greater extent with the 16-base T-oligo. **(c) **SCID mice inoculated intravenously with MCF-7 cells received intravenous T-oligo or diluent injections as per the Materials and methods. Doses giving comparable growth inhibition at 96 hours were used: 20 μM 16mer versus 40 μM 11mer, equivalent to 60 versus 120 nmoles injected into the estimated murine 1.5 ml peripheral blood volume. Both T-oligos increased survival of the mice, with the 16mer having a greater effect.

On-going work in our laboratory has established that many T-oligos exert anti-cancer effects *in vitro *and/or *in vivo*, with molar efficacy determined by several features [18]. To examine T-oligo effects *in vivo*, we selected a 16-base T-oligo and first compared its *in vitro *efficacy to that of the original 11-base T-oligo. At lower concentrations (10 μM and 20 μM), the 16-base T-oligo inhibited MCF-7 yield significantly more than the 11-base T-oligo at 96 hours (64 ± 0.01% versus 31 ± 0.09%, 10 μM, *p *< 0.001; 74 ± 0.01% versus 45 ± 0.19%, 20 μM, *p *< 0.04), although inhibition at 40 μM was comparable (73 ± 0.01% versus 64 ± 0.03%, *p *= 0.25) (Figure 8a). In addition, at 20 μM the 16-base T-oligo induced substantially more p53 and H2AX phosphorylation than the 11-base T-oligo within 48 hours (Figure 8b), demonstrating a higher molar efficacy for the 16-base T-oligo compared to the 11-base T-oligo on MCF-7 cells *in vitro*. Doses of roughly equivalent molar efficacy were selected for comparison in the mouse model.

We then injected MCF-7 cells into the tail vein of SCID mice, a protocol expected to yield tumors in multiple organs and to kill the mice [54]. After 3 days and 31 days, when the first mouse died (a diluent control but still blinded to the investigators), all remaining mice were treated with diluent alone or amounts of T-oligo calculated to yield peripheral blood concentrations of 40 μM (11mer) or 20 μM (16mer) if instantly diluted into the animals total blood volume: 120 nmoles or approximately 23 mg/kg per injection of the 11-base T-oligo or 60 nmoles or 15 mg/kg of the 16-base T-oligo. Injections were given twice daily for 5 days for a total of 20 injections. As expected, between 3 and 6 weeks, all controls either died or had to be sacrificed because of rapid weight loss. Mice treated with the 11mer had statistically longer survival (*p *< 0.007), with 1 of 5 remaining healthy through at least 30 weeks, and 75% of mice treated with the 16-base T-oligo survived without weight loss or other evidence of disease through 30 weeks (*p *< 0.01, control versus 16-base T-oligo), when the experiment was terminated (Figure 8c). Autopsies of the 11mer-treated mice that died at 6 to 8 weeks and the 16mer-treated mice that were sacrificed on the 30th week of the experiment revealed macroscopic tumor deposits similar to those in the diluent-treated mice at their time of death, on average 2 and 28 weeks earlier, respectively. Histological analysis of several tissues, including bone marrow, liver, jejunum, brain, lung, kidney and skin, of a SCID mouse injected 24 hours earlier with T-oligo failed to reveal any evidence of T-oligo toxicity.

## Discussion

Our data demonstrate remarkable effects of T-oligo on MCF-7 human breast cancer cells *in vitro *and on xenograft tumor growth *in vivo*. *In vitro*, T-oligo induces growth arrest, followed by apoptosis or senescence. Interestingly, a single dose of the hydrolysable T-oligo, with a half-life in serum-containing medium of approximately four to six hours [55], induces these responses, possibly attributable to a prolonged half-life of telomere homolog oligonucleotides once in the nucleus [56]. Similarly *in vivo*, delivering T-oligos intravenously twice daily for two periods of five days each appears to rescue some mice from otherwise rapidly fatal innocula of tumor cells and to significantly increase survival overall.

We found that T-oligos are taken up by cells and localize to the nucleus within 30 to 60 minutes after addition to culture medium. Interestingly, it was not necessary to use special delivery systems, such as electroporation, and DNA-liposome and DNA-polymer complexes, used in some studies [57]. This is consistent with our own previous studies [9,19] and other investigators who report similar uptake of oligonucleotides in other cell types without the use of special delivery systems [58,59]. Although the precise mechanism of oligonucleotide uptake is unclear, cell surface receptors have been proposed to perform this function [58,60].

T-oligo rapidly decreased the yields of both breast cancer MCF-7 cells and NME cells, compared to actively proliferating controls, likely by inducing an S phase arrest, but appeared to induce apoptosis and senescence only in MCF-7 cells. This differential T-oligo effect is not due to selective uptake, as T-oligo is taken up by normal and malignant cells equally well, but rather to different cellular responses. In this study we show significantly less activation of downstream effectors by T-oligo in normal NME cells compared to MCF-7 malignant cells, and it is possible that, below a certain phosphorylation threshold for p53 and H2AX, apoptosis and senescence do not occur. This result is consistent with differences in response to T-oligo previously observed between normal human melanocytes and malignant melanoma cells [13], normal murine B cells and B cell lymphoma cells [61], and normal human astrocytes and glioblastoma cells [62].

In MCF-7 breast cancer cells, T-oligos activate and induce DNA damage response proteins that cause apoptosis and senescence of cells. Senescence was determined by β-gal activity and failure to incorporate BrdU or to phosphorylate pRb after fresh medium supplementation. Although the otherwise senescent-like MCF-7 cells were arrested in S phase, not in G_0_/G_1_, as observed with fibroblasts serially passaged to senescence and then refractory to cell division despite provision of fresh serum-containing medium [63,64], this is identical to the status of newborn human fibroblasts rendered senescent by prolonged T-oligo treatment. These cells are also in S phase arrest after 7 days but, if provided fresh medium, largely progress over 24 hours to G_0_/G_1_, and then irrevocably arrest [10].

Our model for explaining the T-oligo effect hypothesizes the existence of innate anti-cancer responses in all cells, normally triggered by opening of the telomere loop and exposure of the single-stranded 3' overhang but also by T-oligos that mimic the overhang.

Consistent with our hypothesis, the same molecular pathways leading to apoptosis/senescence are activated by either T-oligo treatment or telomere loop disruption through ectopic expression of TRF2^DN ^in either MCF-7 cells in the present study or in fibroblasts as reported by our group [65] and other investigators [10,66]. While the muted response to T-oligo of normal versus malignant cells has important clinical implications, its basis is not clear. Our model hypothesizes the existence of innate anti-cancer responses intended to prevent further proliferation of dysregulated cells, responses normally triggered by opening of the telomere loop and exposure of the 3' overhang. We further hypothesize that this mechanism is defeated in malignant cells by over-expression of proteins, such as telomerase, that bind and obscure the telomere overhang [8], but that provision of T-oligos replaces the obscured TTAGGG sequence and initiates the DNA damage-like signaling. This hypothesis is consistent with the observations that knock-down of a non-enzymatic component of telomerase (hTER) in malignant cells may lead to their apoptosis within five days without telomere shortening [67,68], and with the recent demonstration that ATM, ATR and their downstream effector DNA damage response proteins are normally recruited to the telomeres during S phase as part of a cellular mechanism that assures genomic integrity [65]. It is unlikely that expression of telomerase, in the absence of genomic lesions and dysregulated growth, is sufficient to cause T-oligo signaling to proceed through induction of apoptosis or senescence, as normal cells known to express telomerase, such as mitogen-stimulated rapidly proliferating lymphocytes, do not undergo apoptosis in response to T-oligo treatment, while syngeneic lymphoma cells do [61].

Also consistent is the observation that knock-down of POT-1, a telomere-specific single-stranded DNA binding protein [69], also elicits apoptosis (in MCF-7 cells) and senescence (in fibroblasts; not reported in MCF-7 cells) associated with telomere loop disruption and degradation of the 3' overhang [70]. In this latter case, because POT-1 associates with TRF2 [70] and TRF2 has been shown, at least *in vitro*, to bind and inhibit ATM [71], it has been suggested that simple removal of a direct inhibitory effect of POT-1/TRF2 complexes on ATM accounts for the observed ATM activation. However, this mechanism could not account for the apparently identical signaling pathways activation by telomerase knockdown [67,68] or treatment with T-oligos that does not result in telomere loop disruption and consequent digestion of the endogenous 3' overhang [9,15]. Furthermore, over-expression of POT-1 abrogates DNA damage responses induced by POT-1 sequestration with TRF2^DN ^[70]. This finding suggests that masking of the TTAGGG repeat sequence by binding of excess POT-1 even after opening of the telomere loop is sufficient to block signaling through ATM and p53, and that exposure of the otherwise concealed TTAGGG repeat sequence after POT-1 knock-down is a more likely causal event. That T-oligos substitute for exposure of the telomere 3' overhang as a stimulus for DNA damage-like signaling is further suggested by the recent demonstration that WRN, the protein mutated in Werner syndrome [72] and known to localize to the telomere and to resolve the telomere loop structure [73,74], is required for T-oligo initiated signaling [15]. Furthermore, T-oligo-induced γH2AX foci, also characteristic of senescent cells [41], form at or immediately adjacent to telomeres [15].

Drug resistance is the major reason for treatment failure in most human cancers [75-78]. While few cancers eventually display an inherent resistance to chemotherapy, the majority of cancers acquire multi-drug resistance as a result of over-expression of the P-glycoprotein encoded by the *MDR-1 *gene that pumps out of the cell several structurally unrelated chemotherapeutic drugs as well as other compounds [79]. Furthermore, in some cancer cells, in addition to its efflux pump action, P-glycoprotein has an anti-apoptotic effect [80,81]. Thus, identifying treatment options for MDR-1 expressing cancers is of utmost importance. Our experiments show that MCF-7 cells resistant to adriamycin (Adr/MCF-7) are sensitive to T-oligo. The same regulatory proteins are activated in MCF-7 cells resistant or not resistant to adriamycin, leading to their apoptosis. Thus, T-oligos constitute a novel class of anti-cancer agents that alone or in combination with conventional drugs may improve treatment responses for breast carcinoma and other cancers.

In the current study we compared the efficacy of an 11 base and a 16 base oligonucleotide with 100% and 81% telomere homology, respectively. Surprisingly, the 16 base T-oligo was more effective on an equimolar basis at inducing H2AX and p53 phosphorylation and decreasing cell yields *in vitro*, and half the dose in nanomoles, it was more effective than the 11mer at enhancing survival of MCF-7-injected mice. Of note, T-oligos require 3'→5' nucleolytic digestion to be active, presumably by the WRN exonuclease and helicase that is required for T-oligo-induced signaling [15]. It is possible that longer T-oligos provide a better substrate for WRN, but additional experiments are required to delineate this point and to optimize the T-oligo as a cancer chemotherapeutic agent.

Nevertheless, both T-oligos demonstrated anti-tumor effects as determined by mice survival when injected intravenously into SCID mice previously injected with MCF-7 cells. T-oligos were given twice a day for only two periods of 5 days, 3 days after MCF-7 inoculation and 31 days after their inoculation, at the time control mice began to die or had to be sacrificed. Although the dosing regimen was arbitrary, it was sufficient to allow survival of 75% of the 16mer-treated animals for 23 weeks after the last treatment and for 30 weeks after inoculation with MCF-7 cells before the experiment was terminated. In contrast, diluent-treated mice did not survive beyond six weeks. The less effective 11mer also increased the survival of the tumor-bearing mice.

Our experiments show that T-oligos are effective against p16-null MCF-7 breast cancer cells as well as adriamycin-resistant P-glycoprotein-expressing cells that additionally have non-functional p53, suggesting that T-oligos may be effective against at least some chemotherapy-resistant tumors. Importantly, T-oligos are effective in a SCID mouse model of metastatic human breast cancer.

## Conclusion

We suggest that T-oligos provide a novel approach to treating human malignancies, including breast cancer. Our data further support the existence of an innate anti-cancer mechanism that is disabled in cancer cells but that can be triggered by telomere homolog oligonucleotides delivered to the nucleus in the form of T-oligos.

## Abbreviations

Ad = adenovirus; ATM = ataxia telangiectasia mutated; ATR = ATM-Rad3-related; BrdU = 5-bromo-2'-deoxyuridine; β-gal = β-galactosidase; FAM = fluorescein phosphoramidite; FBS = fetal bovine serum; GFP = green fluorescent protein; HBSS = Hank's buffered salt solution; MDR = multiple drug resistant; NME = normal mammary epithelial; PBS = phosphate-buffered saline; pRb = retinoblastoma protein; SA = senescence-associated; SCID = severe combined immunodeficient; TRF = telomeric repeat binding factor.

## Competing interests

MY, MSE and BAG have a conflict of interest in that they have equity in the for-profit start-up company SemaCo, created to commercialize the intellectual property arising in the laboratory, which includes pending patents for the use of T-oligos in the treatment of cancer. This conflict of interest is being monitored by the standard process at Boston University, owner of the patents now licensed to SemaCo.

## Authors' contributions

MY designed the experiments and drafted the manuscript. MSE participated in the study design. IP carried out the *in vitro *experiments. JK carried out animal experiments. LHW performed some *in vitro *experiments. KHC provided the adriamycin-resistant MCF-7 cells. BAG conceived of the study and helped to draft the manuscript. MY and MSE contributed equally to this paper.
